# The Effect of Milk Replacer Composition on the Intestinal Microbiota of Pre-ruminant Dairy Calves

**DOI:** 10.3389/fvets.2019.00371

**Published:** 2019-10-24

**Authors:** James Badman, Kristian Daly, Jennifer Kelly, Andrew W. Moran, John Cameron, Ian Watson, John Newbold, Soraya P. Shirazi-Beechey

**Affiliations:** ^1^Epithelial Function and Development Group, Institute of Integrative Biology, University of Liverpool, Liverpool, United Kingdom; ^2^Institute of Veterinary Science, University of Liverpool, Neston, United Kingdom; ^3^Volac International Ltd, Orwell, United Kingdom

**Keywords:** intestine, microbiota, pre-ruminant, dairy calf, milk replacer

## Abstract

The impact of dietary composition and prebiotics, in promoting the growth of beneficial groups of gut bacteria, is increasingly apparent. Using Illumina MiSeq sequencing of bacterial 16S rRNA genes, this study has aimed to characterize and compare the establishment of the gastrointestinal microbiota in dairy calves given two different commercial milk replacer (MR) diets. MR1 and MR2 contain different levels of macronutrients such as protein and fat. Moreover, differences in manufacturing methods infer that MR2 may contain a greater proportion of conjugated milk oligosaccharides (OS), while MR1 contains more free milk OS. A total of 10 dairy calves, five in each group, were assigned to one of the two MR diets. Freshly voided fecal samples were taken at 0, 7, 14, 28, and 49 days after first consumption of milk replacer. The relative abundance of two individual *Bifidobacterium* species, which are known to utilize milk OS, and *Faecalibacterium prausnitzii* were significantly higher at day 7 in the fecal microbiome of calves fed MR2 compared with MR1. These commensal bacteria are widely regarded as probiotic organisms that confer a health benefit on the host. Our findings suggest that the composition of bovine milk replacers can have significant effects on the establishment of the gut microbiota in pre-weaned (neonatal) dairy calves. Better understanding of milk composition-microbiota-host interactions in early life will inform targeted interventions to increase growth and reduce mortality in young animals.

## Introduction

Microbial colonization of the intestine during early life plays an instrumental role in the stimulation of gut function, development and education of the host immune system. These early life events can have long-standing consequences such as facilitating tolerance to environmental exposures or contributing to disease development in later life (1, 2). The development and activity of the post-natal gastrointestinal microbiota is of critical importance to the health, growth, and performance of new-born animals (3, 4) and is profoundly influenced by the composition of the mother's milk (5). Cow's milk contains approximately 87.8% water, 3.9% fat, 3.2% protein, and 4.8% lactose; it also contains variable chain length oligosaccharides (OS), with a prevalence of ~0.7–1.2 g/L (6).

Typically, dairy calves are fed at least 3 l of colostrum within the first 2 h of life, before being separated from the mother within 24 h of birth. They are subsequently fed either consumable whole milk, non-saleable milk, or milk replacer for a period of 6–8 weeks, until weaning. This milk feeding period of calf development is critical to health, well-being, and productivity. The use of whole milk is costly and commercial milk-replacers have proven to be suitable replacements (7).

Milk replacers (MR) are manufactured using either skim milk, derived from butter-making, or whey, obtained from cheese production. The differing MR formulations, with different levels of protein, fat and implied OS content, may be an important factor in the establishment of a beneficial gut microbiota, with OS acting as high potential bioactive feed ingredients (8, 9).

The objective of this study was to characterize and compare differences in the establishment and succession of gut microbiota in neonatal pre-ruminant dairy calves in response to feeding two different milk replacers having differing nutritional composition.

## Materials and Methods

### Animals and Milk Replacer

Male and female Holstein-Friesian dairy calves taken from their mothers immediately after colostrum intake were housed in standard pens (1.5 m^2^; straw bedding) at the University of Liverpool Woodpark Farm. Maternal colostrum was administered within the first 6 h after birth. Two groups, both containing five calves, were maintained on one of two MR diets (supplied by Volac International Ltd, Hertfordshire, UK) for a period of 7 weeks: (Group 1) MR1, with 55% (w/w) of crude protein content, or (Group 2) MR2, with 74% (w/w) of crude protein being derived from whey protein phospholipid concentrate (WPPC) (For composition of MR see Table 1). WPPC is produced using microfiltration to separate the major whey proteins (α-lactalbumin and β-lactoglobulin) from cheese whey. The membranes used for this filtration commonly have pores designed to retain molecules >10 kDa, allowing sugars, small peptides, minerals, and notably free milk OS to be concentrated in the permeate (8). Milk OS that are conjugated to proteins and phospholipids are retained in the WPPC. Each MR was prepared according to the manufacturer's instructions, at a concentration of 150 g/l, and calves were fed 2.5 l twice per day (8.00 a.m. and 5.00 p.m.). Throughout the feeding trial, all calves had free access to water. There were no cases of enteric or metabolic disturbances, with all animals staying healthy throughout the course of the feeding trials. At day 14, all calves were gradually given solid starter feed (Vita Calf Starter, ForFarmers UK Ltd, Bury St. Edmonds, UK) in addition to MR.

**Table 1:** Composition and analysis of milk replacers.

	**MR1**	**MR2**
**COMPOSITION (% OF MR POWDER)**		
Fat-filled whey protein concentrate (13.5% CP)	89.7	–
Fat-filled whey protein concentrate (21.5% CP)	–	92.4
Vegetable fat blend^†^	18.3	13.7
Hydrolysed wheat gluten	2.4	5.6
Soya protein concentrate	6.0	–
Vitamin and mineral premix	0.5	0.5
DL-methionine	0.3	0.3
Lysine HCl	0.5	0.5
Feed additives^‡^	0.4	0.4
**ANALYSIS (% IN MR POWDER)**		
Crude protein	21.8	26.8
Crude oil	18.8	15.9
Ash	7.7	6.7
Crude fiber	0.25	0.015
Moisture	2.2	2.6
pH	5.8	5.8
Calcium	0.75	0.86
Magnesium	0.1	0.11
Sodium	0.58	0.59
Potassium	1.61	1.41
**CONTRIBUTION FROM VITAMIN AND MINERAL PREMIX**		
**(IU/kg MR powder)**		
Vitamin A	25,000	25,000
Vitamin D3	6,000	6,000
Vitamin E	250	250
**(mg/kg MR powder)**		
Copper	10	10
Iodine	0.25	0.25
Iron	80	80
Manganese	30	30
Selenium	0.4	0.4
Zinc	50	50

† Palm oil, coconut oil;

‡ *citric acid, garlic extract, and flow aid*.

### Collection of Samples

Freshly voided fecal samples were taken, whilst extruding from the anus, from all calves at day 0 (after first consumption of milk replacer), 7, 14, 28, and 49, within 20 min of the morning feed. Each sample was placed in labeled aluminum foil and immediately frozen in liquid Nitrogen. Frozen samples were transported in liquid Nitrogen to the laboratory in Liverpool and stored at −80°C until use. No invasive procedures were used.

### Extraction of Bacterial DNA From Fecal Samples

Bacterial DNA was extracted from frozen fecal samples using the Quick-DNA Fecal/Soil Microbe Miniprep Kit (Zymo Research, Irvine, California, USA) following the manufacturer's guidelines. Purified DNA was quantified using the Quant-iT PicoGreen dsDNA Assay Kit (Life Technologies Ltd, Paisley, UK), with integrity evaluated using agarose gel electrophoresis. Rapid freezing of samples in liquid Nitrogen, followed by a single extraction procedure, is an effective method for preserving intact microbial DNA. This method avoids repeated freeze-thawing of samples, which could be detrimental to the preservation of DNA from gram-negative bacteria (10, 11).

### PCR Amplification of Bacterial 16S rRNA Genes and Illumina MiSeq V4 Sequencing

The amplification of the hypervariable V4-region of the 16S rRNA gene from extracted DNA was achieved using the universal forward and reverse bacterial primers 515f and 806r (12), with the required Illumina flowcell adaptor sequences. Each reverse primer also contained a unique 12 base Golay barcode allowing all samples to be multiplexed (13). To limit PCR-associated bias, each sample was amplified in triplicate and PCR cycling was kept to a maximum of 25 cycles. Each reaction mix contained 1 × Q5 Reaction Buffer (New England Biolabs, Hitchin, Hertfordshire, UK), 0.5 μM of each primer, 0.5 U of Q5 Hot Start High-Fidelity DNA Polymerase (New England Biolabs), and 5 ng template DNA in a final volume of 25 μl. PCR cycling was carried out as follows: initial denaturation at 95°C for 60 s, 25 cycles of denaturation at 95°C for 10 s, annealing at 53°C for 20 s, and extension at 72°C for 15 s, followed by a final extension step at 72°C for 2 min. No template controls were used alongside every sample. Purification of PCR reactions was then performed by agarose gel electrophoresis using the QIAquick Gel Extraction kit, according to the manufacturers' instructions (Qiagen Ltd, Manchester, UK). Purified amplicons were quantified using the Quant-iT PicoGreen dsDNA Assay Kit (Life Technologies Ltd), pooled in equimolar amounts, and sequenced on the Illumina MiSeq platform at the Center for Genomic Research (CGR), Institute of Integrative Biology (IIB), University of Liverpool, UK.

### Analysis of Illumina MiSeq Sequence Reads

Raw sequencing reads were subjected to a strict read-filtering pipeline at the CGR to remove Illumina adaptor sequences (Cutadapt, version 1.2.1) (14), any low-quality bases and reads below 10-bp in length (Sickle, version 1.33) (15). Filtered de-multiplexed reads were then analyzed using the Quantitative Insights into Microbial Ecology 2 (QIIME2) software package (versions 2018.2 and 2018.4, https://qiime2.org) (16). QIIME2's “DADA2” plugin was used to resolve reads to high-resolution amplicon sequence variants (ASVs), which represent, as closely as possible, the original biological sequence of the sequenced amplicon (17). Using sequence quality plots as guidance, the following parameters were used as input for DADA2: --p-trunc-len-f 220 and --p-trunc-len-f 210.

Multiple sequence alignment of ASV representative sequences was carried out using MAFFT software (18). FastTree (19) software was then used to infer unrooted and subsequently rooted maximum-likelihood phylogenetic trees representing the phylogenetic relatedness of ASVs (QIIME2 phylogeny plugin). ASVs were taxonomically classified using a downloaded Naïve-Bayes classifier, pre-trained on Greengenes 13_8 (QIIME2 feature-classifier plugin) (20). Following taxonomic classification, ASVs comprising <10 reads, found in only one sample or classified as Mitochondria or Chloroplast were removed.

### Alpha/Beta Diversity Analyses

Alpha and beta diversity analysis was carried out using an even read depth/subsampling depth of 72,000. Microbial diversity was assessed by calculating the following alpha diversity metrics; Shannon's Diversity Index (Shannon's DI), Faith's Phylogenetic Diversity (Faith's PD), and observed ASVs. Evenness (a comparison of the relative abundance of each species in different samples) was calculated using Pielou's Evenness (Pielou's E). Compositional similarity/dissimilarity between samples (beta diversity) was estimated by generating weighted and unweighted UniFrac, Jaccard and Bray-Curtis dissimilarity matrices for all pairwise sample comparisons. Compositional dissimilarity of samples was visualized using principal co-ordinates analysis (PCoA) of beta-diversity distance matrices. To test for significant associations between alpha diversity metrics and categorical metadata groups (Milk replacer diet, Gender, Subject ID), non-parametric Kruskal-Wallis with Benjamin-Hochberg multiple test correction was applied, whilst significant associations between numerical metadata groups (sampling time-point) and alpha diversity metrics were tested using Spearman's correlation tests. Pairwise comparison of beta diversity distances between categorical metadata groups was analyzed employing permutational multivariate analysis of variance (PERMANOVA), whilst significant correlations between numerical metadata categories and beta diversity distances were investigated using Mantel tests with 1,000 permutations (all listed above performed using QIIME2 diversity plugin).

To test for associations between longitudinal changes in alpha and beta diversity over time and for the different milk replacer diets we performed linear mixed-effects (LME) regression analysis. This accounted for subject-specific variation by using subject ID as a random effect, whilst allowing identification of longitudinal differences in alpha/beta diversity due to milk replacer diet by using that category as a fixed effect. One-way ANOVA with Tukey's multiple comparison test or Student's unpaired *t*-test (GraphPad Prism 7.05; GraphPad Software, La Jolla, CA, USA) were used to identify taxa that were significantly associated with each milk replacer diet and those showing significant changes in abundance over time. Results were considered significant if *p* < 0·05.

### Quantitative Real-Time PCR Analysis of Microbial Abundance

The determination of total bacterial numbers in DNA extracted from calf fecal samples was achieved by quantitative real-time PCR (qPCR) using a Rotorgene 3000 (Qiagen Ltd) and SYBR Green JumpStart Taq ReadyMix (Sigma Aldrich Ltd, Crawley, Sussex, UK). Determination of total 16S rRNA gene copy number, achieved by quantification of bacterial DNA amplified using eubacterial 16S rRNA primers, in comparison to standard curves constructed from a reference plasmid, was used as a measurement of total bacterial numbers (21). All assays were performed in triplicate using the following parameters: initial denaturation at 95°C for 2 min followed by 40 cycles of 95°C for 15 s, 53°C for 15 s, and 72°C for 30 s. Calculation of 16S rDNA copy number was achieved using Rotor-Gene 3000 quantification software.

## Results

Using DNA extracted from freshly-voided feces of neonatal calves maintained on milk replacer, and Illumina MiSeq sequencing of 16S rRNA gene V4 amplicons, our aim was to uncover community structure, diversity and succession of the gastrointestinal microbiota over the first few weeks of life in calves consuming MR1 and MR2.

**Figure 1:**
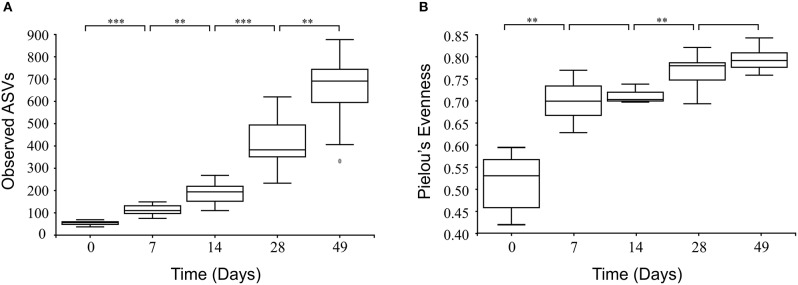
Box and whisker plots showing level of microbial diversity **(A)** and evenness **(B)** of the calf fecal microbiome over time. Boxplots display the median as the middle line whilst the perimeters of the box display the 1st and 3rd quantiles of the data. The whiskers extend to the highest and lowest values. ^**^p < 0.01; ^***^p < 0.001.

**Figure 2:**
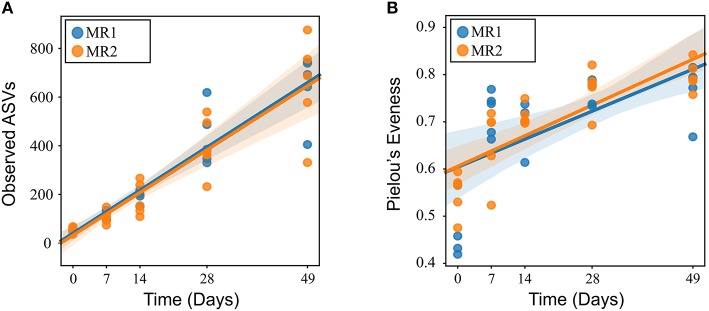
Linear mixed effects regression analysis of microbial diversity **(A)** and evenness **(B)** of the calf fecal microbiome using time as a continuous variable and calf ID as a random effect.

**Figure 3:**
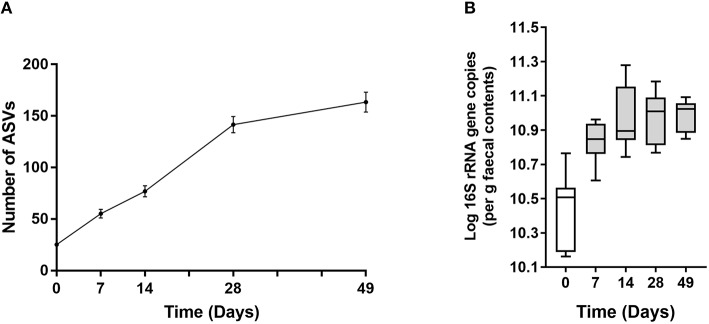
**(A)** Line plot showing increase in number of ASVs over time. Values are mean ± SEM. **(B)** Total 16S rRNA gene copy number in fecal samples over time. Boxplots display the median as the middle line whilst the perimeters of the box display the 1st and 3rd quantiles of the data. The whiskers extend to the highest and lowest values. Gray bars indicate significance (*p* < 0.001) compared with day 0.

**Figure 4:**
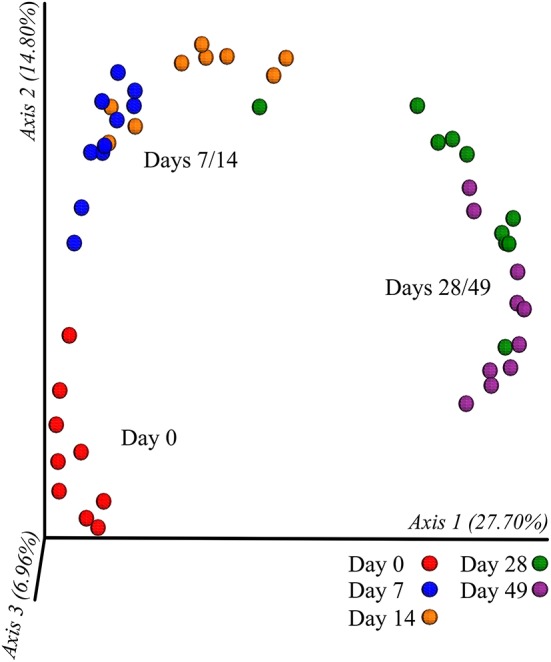
Clustering of calf fecal microbiome over time based on principal co-ordinates analysis (PCoA) of Jaccard dissimilarity measures. Plotted using the first three principal co-ordinates accounting for ~50% of the observed variation. Samples are colored by individual sampling days and labeled by indicated cluster (day 0, days 7/14, and days 28/49).

In all calves, regardless of milk replacer, microbial diversity significantly increased between each sampling time point (*p* < 0.05; Figure 1A), whilst the level of microbial evenness increased significantly between days 0 and 7 and days 14 and 28 (*p* < 0.05; Figure 1B). Both microbial diversity and evenness showed a significant positive association with time in all calves, indicating an increase of overall microbial diversity over the period of the dietary experiment (Figures 2A,B). This enhancement in diversity is also demonstrated by the increasing numbers of distinct ASVs (minimum 0.1% of total) at each time point; the fecal microbiome of each calf at day 0 comprising an average of 25 ASVs, and at day 49 containing an average of 163 ASVs (Figure 3A). QPCR analysis showed a significant increase in total bacterial numbers immediately after birth, with considerably higher microbial density in days 7–49 compared with day 0 (*p* < 0.05) (Figure 3B). Beta diversity analysis of the fecal microbiome of all calves revealed a significant change in composition between each successive time point (*p* < 0.05; PERMANOVA). Moreover, the level of microbial dissimilarity was positively correlated with time, indicating that microbial composition became significantly more divergent throughout the sampling period (*p* < 0.01; Mantel test, Spearmans rho correlation). A stronger correlation was found using qualitative (unweighted UniFrac; *r* = 0.77) compared with quantitative dissimilarity measures (weighted UniFrac; *r* = 0.27), showing that compositional changes over time were more related to the emergence of new species into the community (presence/absence) rather than changes in abundance of existing community members. Furthermore, principal coordinates analysis (PCoA) of Jaccard dissimilarity revealed that the fecal microbiota of all calves grouped into three distinct clusters: day 0, days 7 and 14, and days 28 and 49. This demonstrated that samples within each of these three clusters show similar microbial community composition (Figure 4), but also the divergence of the microbial communities from the previous time period.

**Figure 5:**
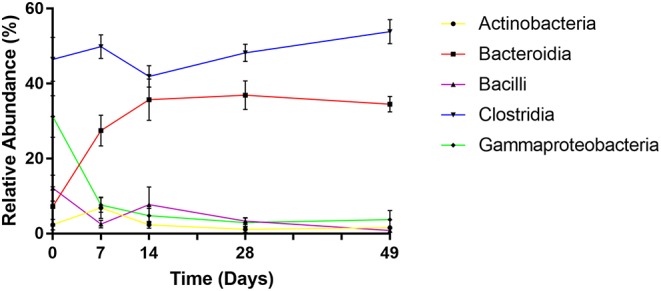
Changes in relative abundance of dominant bacterial classes over time. Values are mean ± SEM.

Phylogenetic classification demonstrated that bacterial communities were dominated by five main bacterial classes: *Actinobacteria, Bacteroidia, Bacilli, Clostridia*, and *Gammaproteobacteria* comprising average relative abundances of 3, 28, 5, 48, and 10% of total, respectively. However, despite being ubiquitously present, each of these classes displayed distinct abundance profiles over time (Figure 5).

At day 0 the fecal microbiome was dominated by *Clostridia* (mean 46%) and *Gammaproteobacteria* (mean 31%). Abundance of *Bacilli, Bacteroidia*, and *Actinobacteria* were substantially lower (mean 12, 7, and 2%, respectively). By day 7 however, levels of *Gammaproteobacteria*, exclusively represented by *Enterobacteriaceae*, had declined dramatically (to a mean abundance of 8%) (*p* < 0.001) before reducing further to a steady-state abundance from day 14 onwards (mean 3–5%). *Bacteroidia* displayed an opposite profile, exhibiting lower abundance at day 0 (mean 7%), comprising predominantly of *Bacteriodaceae*, before rapidly increasing to mean abundances of 27, 36, 37, and 35% of total at subsequent sampling points (*p* < 0.001), attributable to the emergence of *Prevotellaceae* and *Paraprevotellaceae*. *Bacilli* abundance decreased to <1% by day 49, due to decline of *Streptococcaceae* (*p* < 0.01). *Actinobacteria*, with a mean abundance of 2% at day 0, peaked at 7% at day 7 due to increased *Bifidobacteriaceae*, before returning to an average level of 1–2% by day 28. Mean levels of *Clostridia* remained stable between 51 and 59% of total throughout the experiment. However, during successive time points, there was marked decline in the abundance of *Clostridiaceae* and increases in *Lachnospiraceae, Ruminococcaceae*, and *Veillonellaceae*. Notably, *Faecalibacterium prausnitzii*, which was undetected at day 0 rose to mean 19% of total by day 7 (*p* < 0.001).

**Figure 6:**
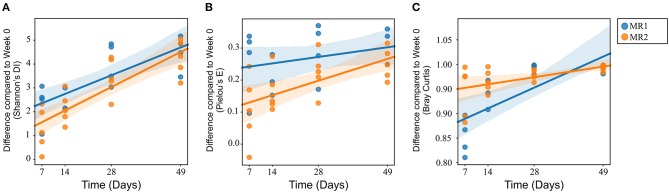
Linear mixed effects regression analysis of the change in microbial diversity (**A**, *p* = 0.07), evenness (**B**, *p* < 0.01), and composition (**C**, *p* < 0.001) compared to week 0 (baseline) for each milk replacer. Time is used as a continuous variable, milk replacer as a fixed effect and calf ID as a random effect.

There were no significant differences in alpha diversity or microbial evenness of the microbiome of calves fed either MR1 or MR2 (Figures 2A,B). However, a difference in the rate of accumulation of diversity and evenness between calves fed either MR1 or MR2 was seen. Using day 0 as the baseline, regression analysis revealed that levels of microbial diversity (*p* = 0.07) and evenness (*p* < 0.01) increased at a faster rate in calves fed MR1, compared with calves fed MR2 (Figures 6A,B). By comparing the change in microbial composition from day 0 to each subsequent time point, we noted that MR1-feeding resulted in a greater change in composition compared to day 0 than did MR2 (*p* < 0.001), meaning by day 49, the composition of the microbiome in MR1-fed calves was significantly more dissimilar to day 0 than with MR2 (Figure 6C). This corresponds to the differences observed in alpha diversity, which demonstrated that MR1-fed calves accumulated a greater degree of diversity and evenness when compared to day 0 than did MR2-fed calves (Figures 6A,B).

**Figure 7:**
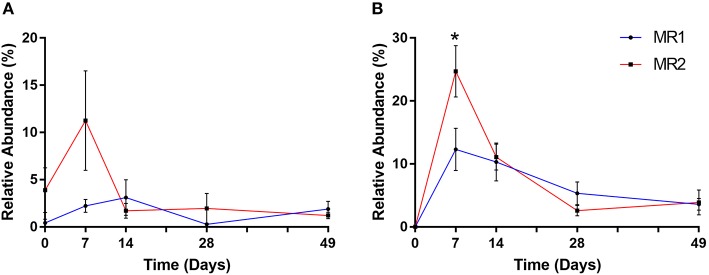
Changes in relative abundance of *Bifidobacterium* **(A)** and *Faecalibacterium prausnitzii* **(B)** over time. Values are mean ± SEM. ^*^p < 0.05.

After the first week of MR feeding, it was notable that two prominent gut bacterial populations, *Bifidobacteriaceae* and *Ruminococcaceae*, were substantially more abundant in MR2- than MR1 fed calves. In the case of *Bifidobacteriaceae*, the mean relative abundance at day 7 was 2% with MR1, but over 11% of total with MR2 (Figure 7A). ASV level analysis revealed that two individual *Bifidobacterium*-classified ASVs (with 100% sequence identity matches to multiple *Bifidobacterium* species) accounted for this increased abundance in MR2-fed calves. One individual ASV identified as *Faecalibacterium prausnitzii*, responsible for the dramatic increase in abundance of *Ruminococcaceae*, was undetectable at day 0. However, the mean population abundance of this bacterial species increased to 24% in MR2-fed calves by day 7 compared to 12% with MR1 (*p* < 0.05) (Figure 7B). By day 49 however, levels of *Bifidobacterium* and *F. prausnitzii* had declined to below 2 and 4% of total, respectively, for both diets (Figures 7A,B).

## Discussion

The assembly of microbial communities within the gastrointestinal tract during early life plays a critical role in maturation of the endocrine, mucosal immune and central nervous systems, strongly influencing and supporting health, and well-being of the young. Disruption of this optimal bacterial succession can contribute to life-long deficits in growth, development, and immunity (1, 2). Thus, characterizing the colonization and succession of the gut microbiota is of great importance to our understanding of host-microbiome interactions in this crucial post-natal phase.

In this study we demonstrate that the composition, structure and diversity of the calf fecal-associated microbiota, proposed as representing the distal colonic microbiota, is highly dynamic with distinct microbial populations present at birth and throughout the first 7 weeks of life, likely due to changing physicochemical conditions and substrate availability in the gut (22).

Our results show that the intestinal microbiota at birth is characterized by low diversity and a relative dominance of the phyla *Proteobacteria*, most notably *Enterobacteriaceae*. These first colonizers are facultative anaerobes that can respire oxygen, thus creating and maintaining enteric anoxia (23). This anaerobic environment is perfect for the establishment of bacterial groups such as *Actinobacteria, Bacteroidetes*, and *Firmicutes* which thrive in such conditions. We show here that throughout the first few weeks of life there is rapid succession and emergence of diverse species in the neonatal calf gut microbiome. Whilst during this period *Proteobacteria* levels decline rapidly, the arrival and dominance of *Firmicutes* and *Bacteroidetes* increases over time. The distinct transitions in microbiome composition, between day 0, days 7 and 14, and days 28 and 49, likely reflect adaptation and succession of the gut microbiota, initially to the intake of milk replacer from day 0, and later to the additional substrates available following intake of solid starter feed at day 28.

There were no substantial differences in the overall diversity or evenness of the microbiome between calves fed either MR1 or MR2. However, by using day 0 as the baseline we demonstrate that the diversity and succession of the calf intestinal microbiota is differentially influenced by each respective MR. The results showing that the rate of change in microbial diversity was greater in calves fed MR1 indicates that macronutrient composition of this MR may contain more widely utilized substrates for microbial fermentation, encouraging the development of a broader, and more diverse microbiome compared to the initial community, and promoting the emergence of novel species. In contrast, MR2 may contain substrates that are only available to a relatively narrow range of microbial populations, thereby having a greater influence on species already present in the community and achieving a stable microbiota more quickly.

Furthermore, when calves were fed with MR2, we observed an enhanced abundance of two key intestinal bacterial groups, *Bifidobacteriaceae* and *Ruminococcaceae*, and specifically of two individual *Bifidobacterium* species and *F. prausnitzii*, in comparison with MR1-feeding.

There are compositional differences in the macronutrient content of these MR, making it difficult to identify specific nutrient(s) that are modifying the microbiota. However, it has been shown that *Bifidobacterium* are uniquely able to comprehensively ferment milk OS as primary substrates (24, 25). This ability bestows a significant competitive advantage on *Bifidobacterium* species during this pre-weaning period when milk is the primary diet (26, 27). Milk OS have been shown to be a key factor in the selective enrichment of intestinal bacteria in newborn humans (28) and despite the levels of OS in bovine milk being much lower than in human milk (9), the observed enrichment of this specialized bacterial group infers that milk OS present in these MR may be influencing the gut microbiome. Although we have no precise data on the amount or type of OS in each MR, differences in manufacturing proposes that MR2 contains a greater proportion of conjugated milk OS due to the higher levels of WPPC, while MR1 is expected to contain more free milk OS due to the higher inclusion of whey permeate. *Bifidobacterium* are known to have pronounced beneficial effects on physiological conditions within the gut, aiding intestinal development and helping to prevent intestinal dysbiosis (29).

The presence and activity of *F. prausnitzii*, a common gut inhabitant known to cross-feed with *Bifidobacteria* (30), is also regarded as being beneficial to host health. In pre-weaned dairy calves, the prevalence of *F. prausnitzii* in the first week of life has been positively associated with increased weight gain and reduced incidence of diarrhea (31).

It is notable that for both *Bifidobacterium* and *F. prausnitzii*, population levels peaked at day 7 following the first consumption of MR, and these levels were not sustained over the course of the trial. This observation suggests that these species are uniquely adapted to react rapidly to the initial influx of substrates present in MR before succession occurs over time.

Diet and dietary supplements play a major role in the development of the gut microbiome (10, 32). This study provides a novel insight into the effects of milk replacer on the gastrointestinal microbiota of new-born dairy calves, and suggests that differences in nutritional composition, can have a major impact on microbiota composition, diversity and succession in pre-weaned calves. It is apparent that post-natal colonization of the gastrointestinal tract is profoundly influential, not only in terms of gut health but also for the health of the whole animal. Thus, the milk-microbiota interaction may act as a primary intervention target for optimizing growth and health in young animals.

## Data Availability Statement

Illumina 16S rRNA gene sequence data has been deposited in the European Nucleotide Archive (ENA) under study accession number PRJEB33625.

## Ethics Statement

Ethical review and approval was not required for the animal study because use of fecal samples is approved by the UK Home Office as a non-invasive procedure requiring no approvals.

## Author Contributions

SS-B conceived and designed the experiments. JB, KD, and AM performed the experiments. JK performed bioinformatic analysis. JC, JN, and IW provided nutritional advice. SS-B, JB, and KD wrote and revised the manuscript. All authors reviewed the manuscript.

### Conflict of Interest

JN and IW were employed by Volac International Ltd. Volac International Ltd was not involved in the design, execution, or interpretation of data. The remaining authors declare that the research was conducted in the absence of any commercial or financial relationships that could be construed as a potential conflict of interest.
